# Antioxidant Properties and Antioxidant Compounds of Various Extracts from the Edible Basidiomycete *Grifola Frondosa *(Maitake)

**DOI:** 10.3390/molecules16043197

**Published:** 2011-04-15

**Authors:** Jan-Ying Yeh, Li-Hui Hsieh, Kaun-Tzer Wu, Cheng-Fang Tsai

**Affiliations:** 1 Department of Biotechnology, Asia University, Wufeng, Taichung County 41354, Taiwan; 2 Department of Plant Pathology, Agricultural Research Institute, Wufeng, Taichung County 41301, Taiwan

**Keywords:** antioxidant property, antioxidant components, *Grifola frondosa*, mushroom

## Abstract

*Grifola frondosa* is an edible mushroom currently available in Taiwan. Ethanolic, cold-water and hot-water extracts were prepared and their antioxidant properties were investigated. At 1 mg/mL, *G. frondosa* T1 and T2 cold-water extracts showed high reducing powers of 1.02 and 0.50, respectively. Chelating abilities on ferrous ions of *G. frondosa* T1 and T2 were higher for cold-water extracts than for ethanolic and hot-water extracts. For the scavenging ability on 1,1-diphenyl-2-picrylhydrazyl (DPPH) radical, *G. frondosa* T1 and T2 extracts were effective in the following order: ethanolic > hot-water > cold-water. The *G. frondosa* hot-water extract showed high scavenging ability on superoxide anions. Total phenols, flavonoids, ascorbic acid and α-tocopherol are the major antioxidant components found in the various *G. frondosa* extracts. Based on EC_50_ values (<20 mg/mL) obtained, the various extracts from *G. frondosa* investigated in this study display potent antioxidative properties.

## 1. Introduction

Oxidation is essential to many organisms for the production of energy to fuel biological processes. However, the uncontrolled production of oxygen-derived free radicals is involved in the onset of many diseases such as cancer, rheumatoid arthritis, and atherosclerosis, as well as in the degenerative processes associated with aging [1]. In order to reduce free radical damage to the human body, synthetic antioxidants are used for industrial processing at the present time. However, the most commonly used synthetic antioxidants have been suspected of being responsible for liver damage and carcinogenesis [2]. Thus, it is essential to develop and utilize effective and natural antioxidants that can protect the human body from free radicals and retard the progress of many chronic diseases.

One of the old Chinese proverbs, “Medicine and foods have a common origin”, is quite relevant to those people who consume phytoceuticals as dietary supplements. Mushrooms are one of such food items that health-conscious people enjoy. They have been used as both food and medicine through recorded history and are one of the natural sources of physiologically active compounds that have been studied for the development of both natural medicine and pharmaceutical products.

*G. frondosa* (maitake) a basidiomycete fungus belonging to the *Polyporaceae *family, is widely used in Japan, China and Korea as a traditional food additive [3]. Maitake literally means “dancing mushroom”. Reportedly people who found the mushroom in deep mountain valleys started dancing with joy since they knew its delicious taste and the health benefits. Maitake was, and still is, one of the most valuable and expensive mushrooms [4].

Recently, various bioactive properties of this mushroom have been explored, thus attracting considerable attention around the globe. The fruit body and liquid-cultured mycelium of this mushroom have been reported to contain useful anti-tumor polysaccharides. These polysaccharide have been identified as many types of glucans (e.g., β-1,6 and β-1,3-) [5]. Polysaccharides obtained from *G. frondosa *have demonstrated many interesting biological activities, including anti-tumor [5], anti-hypertensive [6] anti-diabetic [3], and anti-hyperliposis effects [7]. It is reported that D-Fraction, a polysaccharide extracted from the maitake mushroom activates macrophages, dendritic cells, and T cells, inhibits tumor cell growth, enhances the cytotoxicity of NK cells by inducing the production of IL-12, and improves the symptoms and secondary diseases caused by HIV [8].

Although research has focused mainly on the therapeutic effects and cultivation methods of this edible mushroom, little information is available regarding to its antioxidative properties. Our objective was to investigate the antioxidant properties of ethanolic, cold-water and hot-water extracts from *G. frondosa* fruit bodies, including reducing power, scavenging ability on DPPH and superoxide anion, and chelating ability on ferrous ion. The contents of potential antioxidant components in these extracts were also determined.

## 2. Results and Discussion

### 2.1. Extraction Yields

The yields of water extracts were significantly higher than that of ethanolic extracts for the samples from the same strain. *G. frondosa* T1 and T2 extract yields were in the descending order of cold water (62.6% and 59.6%, respectively) > hot water (53.1% and 50.8%, respectively) > ethanol (17.1% and 18.6%, respectively). The discrepancy in the yields for water and ethanolic extracts may be due to the fact that water extracts contain more water-soluble components, such as soluble polysaccharides, which may precipitate from aqueous suspension of fruit bodies by ethanol extraction [9]. The yield for cold water extraction was higher than the hot water extraction. Other study also found that the yield of cold water extract was higher than those of hot water extract for *Pleurotus citrinopileatus *fruit bodies [10].

### 2.2. Reducing Power

**Figure 1:**
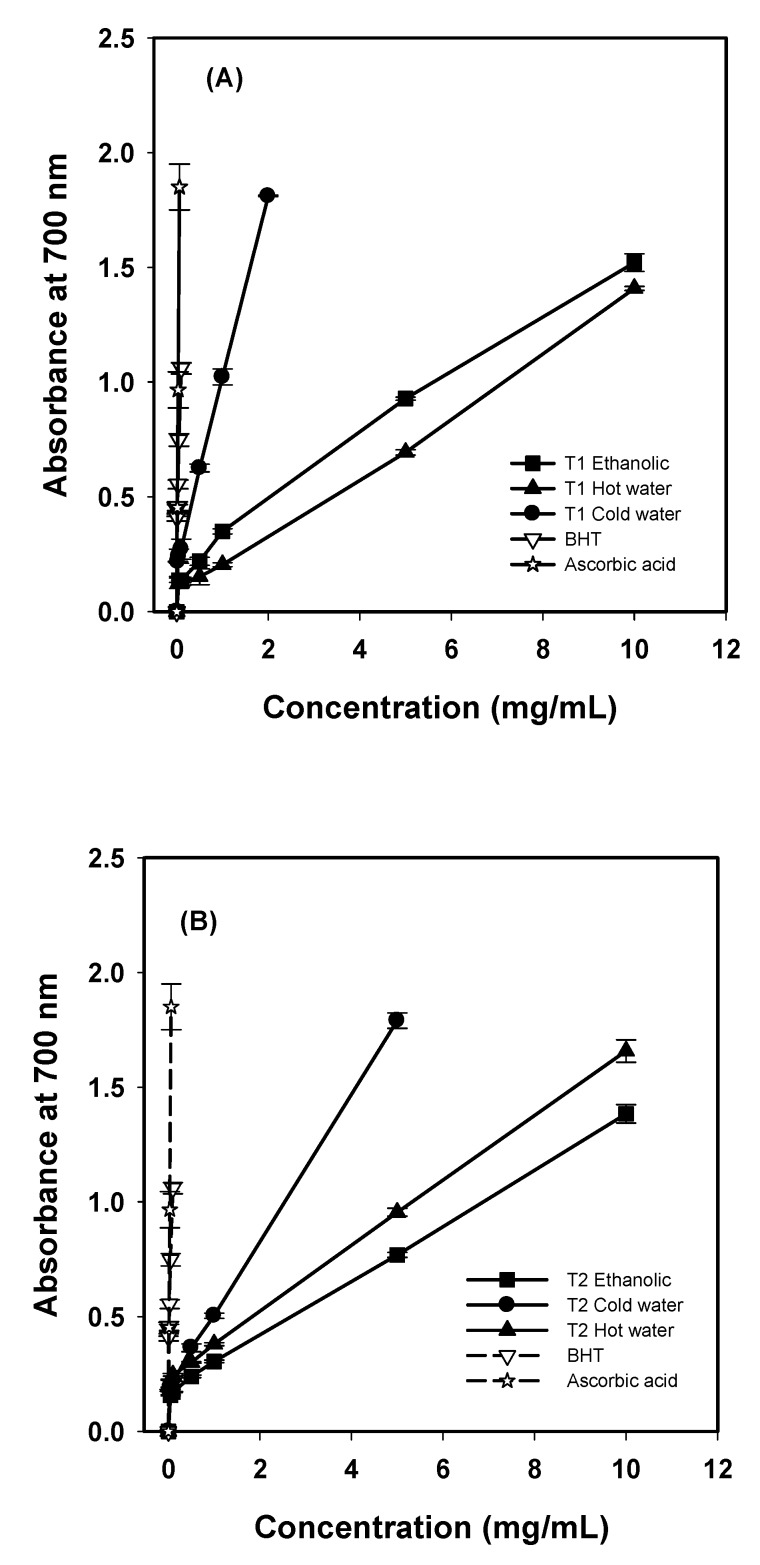
Reducing power of various extracts from *G. frondosa *T1 (A), and *G. frondosa *T2 (B). Each value is expressed as mean ± S.D. (n = 3).

The reducing power assay determines the electron-donating ability of antioxidants using the potassium ferricyanide reduction method. The presence of reducers in the test solution leads to the reduction of the Fe^3^^+^/ferricyanide complex to the ferrous form. The reducing capacity of a compound may serve as a significant indicator of its potential antioxidant activity. The reducing powers of three extracts from *G. frondosa* T1 and T2 increased rapidly with the increase in concentration, as shown in Figure 1. 

The reducing powers of cold-water extracts from both *G. frondosa* T1 and T2 were excellent. At 1 mg/mL, the reducing powers of cold-water extracts T1 and T2 were 1.02 and 0.50, respectively, higher than that of ethanolic (0.35 and 0.30 for *G. frondosa* T1 and T2, respectively) and hot-water extracts (0.20 and 0.38 for *G. frondosa* T1 and T2, respectively). The reducing powers of *G. frondosa* T1 and T2 were 1.81 at 2 mg/mL and 1.75 at 5 mg/mL, respectively, and were higher than that of *H. marmoreus* fruit bodies, which is reported to be 0.99 at 5 mg/mL [11]. However, the reducing power of ascorbic acid was 0.97 at 0.03 mg/mL and that of BHT was 1.06 at 0.1 mg/mL.

The reducing power of hot-water extracts from *G. frondosa* T1 and *G. frondosa* T2 was 0.69 and 0.95 at 5 mg/mL, respectively. Mau *et al*. [12] have reported that hot-water extracts of mature and baby Ling chihs revealed the reducing powers of 1.08 and 1.04 at 5 mg/mL, respectively. In addition, the hot-water extract of *Agrocybe cylindracea *strain B exhibited a reducing power of 0.87 at 5 mg/mL [13]. *G. frondosa* T1 and *G. frondosa* T2 hot-water extracts seem to be lower in reducing power, as shown in Figure 1, when compared to those reported by Mau *et al.* [14]. Furthermore, *G. frondosa* T2 hot-water extract was higher than the same extract from *Agrocybe cylindracea *strain B in reducing power.

As to ethanolic extracts, the reducing powers of *G. frondosa* T1 and T2 were 0.93 and 0.77 at 5 mg/mL, respectively. Tsai *et al*. [14] reported that the reducing powers of ethanolic extracts were 0.37, 0.25 and 0.61 at 5 mg/mL for *A. blazei*, *A. cylindracea* and *B. edulis,* respectively. Apparently, the reducing powers of ethanolic extracts from *G. frondosa* T1 and T2 were more effective than those previously reported.

The reducing capacity may be a significant index of antioxidant activity. Various mechanisms related to antioxidant activities were suggested, including chain initiation, binding of transition metal ion catalysts, decomposition of peroxides, prevention of continued hydrogen abstraction, reductive capacity, and radical scavenging [15].

### 2.3. Scavenging Ability on DPPH Radicals

**Figure 2:**
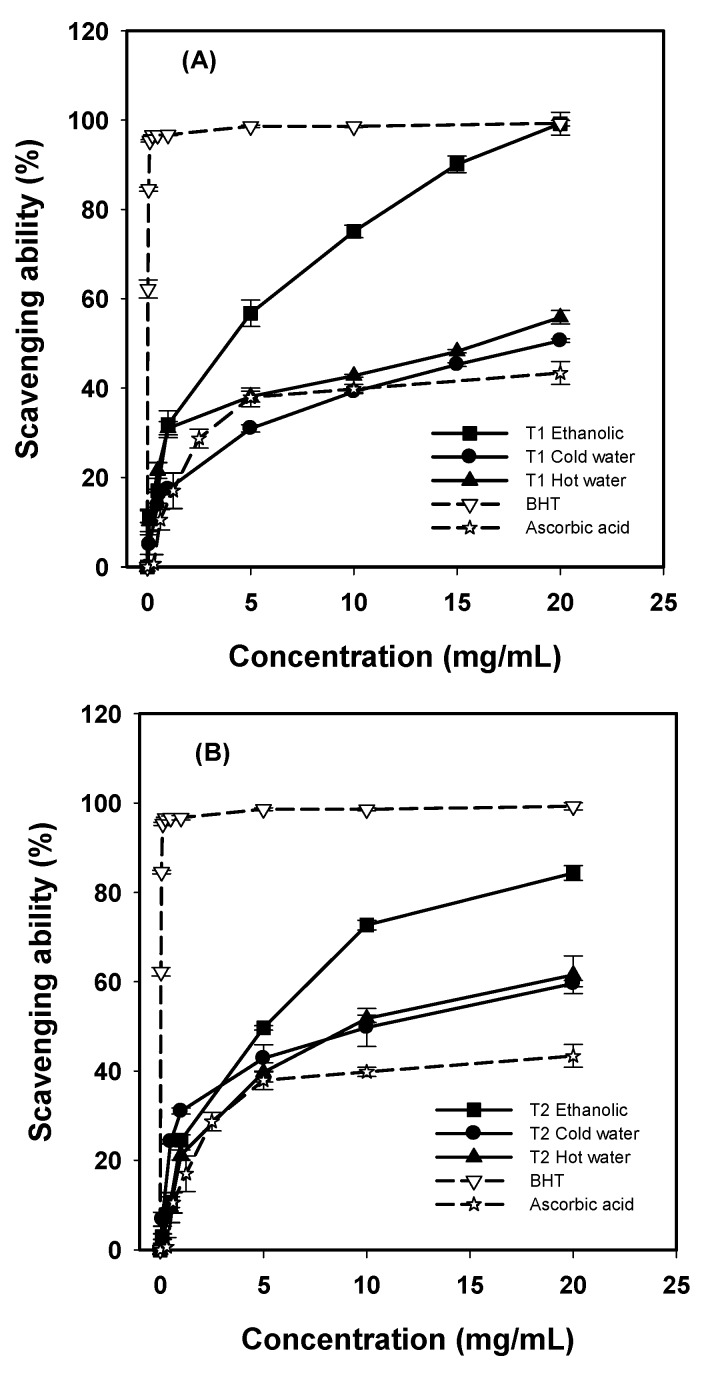
Scavenging ability of various extracts from *G. frondosa *T1 (A), and *G. frondosa *T2 (B) on DPPH radicals. Each value is expressed as mean ± S.D. (n = 3).

Stable DPPH radicals are widely used to evaluate the antioxidant activities of proton-donating substances according to hydrogen donating ability. DPPH radicals accept electrons or hydrogen radicals to form stable diamagnetic molecules. The antioxidant activity of substances can be expressed as the reduction capability of DPPH radical at 517 nm [16]. This chromogen radical compound can directly react with antioxidants and the procedure is simple, rapid and sensitive. Hence, the model of scavenging the stable DPPH radical is a widely used method to evaluate the free radical scavenging ability in variety of samples. Generally, methanolic and/or ethanolic extracts were more effective in scavenging abilities than hot-water extract for most mushroom species reported previously [17]. Similar observations were found in this study. The ethanolic extracts from *G. frondosa* T1 and T2 showed higher scavenging ability on DPPH radicals than water extracts (Figure 2). The ethanolic extracts of *G. frondosa* T1 and T2 scavenged DPPH radicals by 99.19% and 84.36% at 20 mg/mL, respectively. For cold-water extracts, the scavenging abilities of *G. frondosa* T1 and T2 were 50.62% and 59.58% respectively, at 20 mg/mL. However, for the hot-water extracts, the scavenging abilities of *G. frondosa* T1 and T2 was 55.87% and 61.56% respectively, at 20 mg/mL. The BHT had considerably high scavenging ability of 95.34% at 0.125 mg/mL. In addition, ascorbic acid revealed a lower scavenging ability of 43.39% at 20 mg/mL.

At 20 mg/mL, the scavenging abilities of hot-water extracts from mature and baby Ling chih, mycelia on DPPH radicals were 73.8%, 80.1%, and 91.2%, respectively [12]. Hot-water extracts of *A. blazei*, *A. cylindracea *and *B. edulis *revealed scavenging abilities of 51.6%, 39.2% and 53.7%, respectively, at 20 mg/mL [14]. The scavenging ability of hot-water extracts from *G. frondosa* T1 and T2 were higher than that from *A. blazei*, *A. cylindracea *and *B. edulis*, but were less effective than that from mature and baby Ling chih, mycelia.

Tsai *et al*. [14] reported that the scavenging abilities of three ethanolic extracts were 97.4%, 95.8% and 94.3%, respectively, at 20 mg/mL for *A. blazei*, *A. cylindracea* and *B. edulis*. The scavenging ability of ethanolic extract from *G. frondosa* T1 in this study was comparable to that previously reported.

High scavenging ability in ethanolic extract may attribute to high level of antioxidant components in the extract, which react rapidly with DPPH radicals and reduce most DPPH radical molecules. The results from this study indicated that ethanolic extracts were free radical scavengers and behaved possibly as primary antioxidants. Various extracts of *G. frondosa* T1 and T2 may react with free radicals, the major initiators of the autoxidation chain of fat, and accordingly terminate the chain reactions.

### 2.4. Chelating Abilities on Ferrous Ions

The chelating effects of various extracts on Fe^2^^+^ were determined by the formation of ferrozine-Fe^2^^+^ complexes. Chelating agents are able to capture ferrous ion before ferrozine, thus hindering the formation of ferrozine-Fe^2^^+^. Spectrophotometic examination of ferrozine-Fe^2^^+^ absorbance can accordingly be used to calculate ferrous ion chelating activity. The metal chelating capacity is important since it reduces the concentration of transition metals that may act as catalysts to generate the first few radicals to initiate the radical-mediated oxidative chain reactions in biological and/or food systems. Ion chelating agents also may inhibit the Fenton reaction and hydroperoxide decomposition [18].

**Figure 3:**
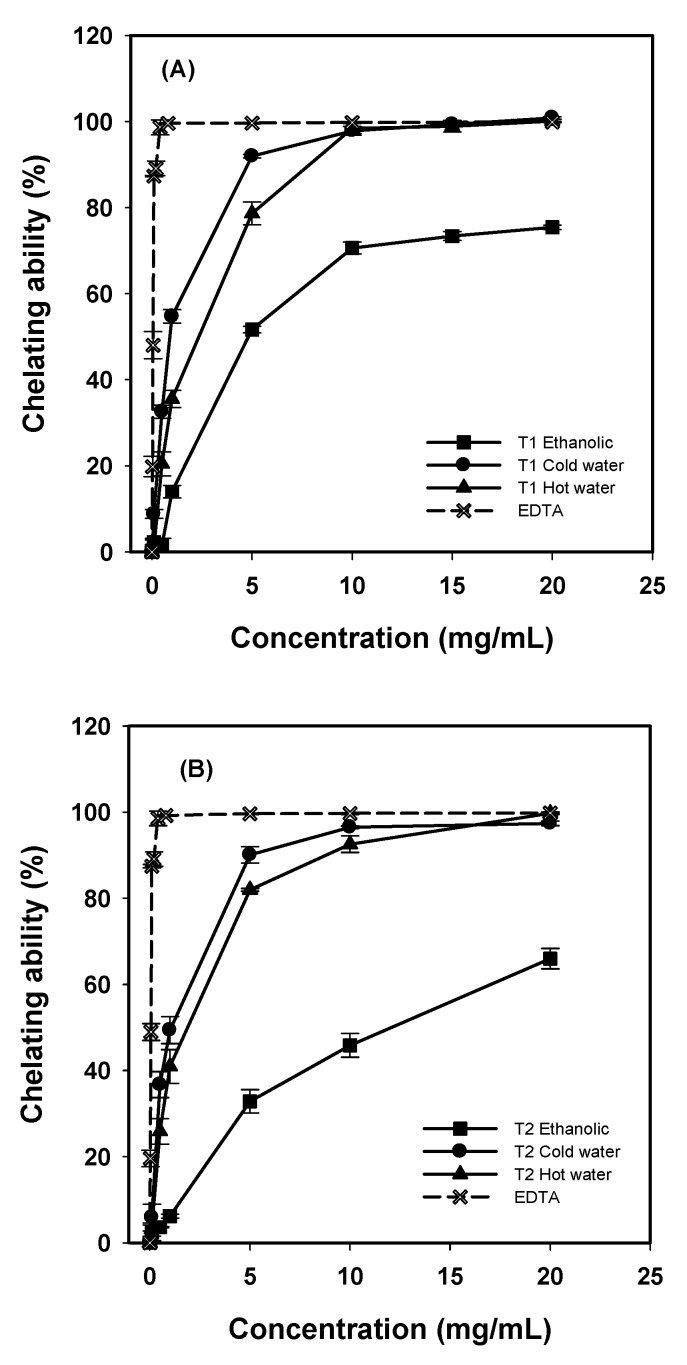
Chelating ability of various extracts from *G. frondosa *T1 (A), and *G. frondosa *T2 (B) on ferrous ions. Each value is expressed as mean ± S.D. (n = 3).

Chelating abilities of various extracts increased as concentration rose (Figure 3). The chelating abilities of water extracts from *G. frondosa* T1 and T2 on ferrous ions were higher than that of ethanolic extracts. At 5–20 mg/mL, chelating abilities were 92.0–100%, 78.7–100%, and 51.7–75.4% for cold-water, hot-water and ethanolic extracts from *G. frondosa* T1, respectively, and 90.1–97.4%, 81.9–99.7%, and 32.8–66.0% for that from *G. frondosa* T2, respectively. Ethylenediaminetetraacetic acid (EDTA) exhibited a high chelating ability of 99.4% at 0.4 mg/mL.

At 5 mg/mL, chelating abilities were 66.6% and 94.1%, respectively, for cold-water extracts from *P. citrinopileatus *and *H. marmoreus* fruit bodies [11]. Apparently, the chelating ability of *G. frondosa* T1 cold-water extract was comparable to that of *H. marmoreus*, and higher than that of *P. citrinopileatus.* Tsai *et al*. [14] reported that chelating abilities of ethanolic extracts were 36.2–58.8%, 24.6–59.8%, and 37.4–61.8% for *A. blazei*, *A. cylindracea* and *B. edulis *at 5–20 mg/mL, respectively. The chelating ability of *G. frondosa* T1 ethanolic extract was obviously more effective than those reported previously. Apparently, the chelating ability of hot-water extracts of *G. frondosa* T1 and T2 were higher than that reported previously. The results in this study suggest that extracts from *G. frondosa* T1 and T2 show the noticeable chelating iron capacity and may act as chelating agents to prevent and/or reduce lipid peroxidation.

### 2.5. Scavenging Effect on Superoxide Anion

Superoxide anion, which is formed in almost all aerobic cells, is a major agent in the mechanism of oxygen toxicity [19]. Oxygen anion (O_2_^•**−**^) is also a highly reactive chemical species and can be generated under regular physiological conditions. In addition to direct attack of important biological molecules, O_2_^•**−**^ may also be involved in the formation of singlet oxygen and hydroxyl radicals, which may increase local oxidative stress and initiate cellular damage and pathological incidents such as arthritis and Alzheimer’s disease. Therefore, to study the scavenging of a superoxide anion is of great importance to human health.

**Figure 4:**
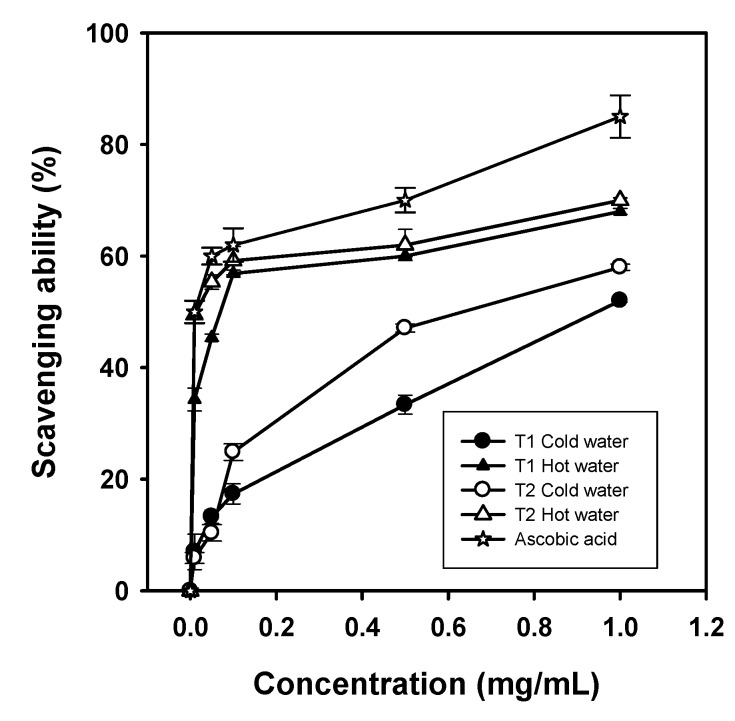
Scavenging ability of cold-water and hot-water extracts from *G. frondosa *T1 and T2 on superoxide radicals. Each value is expressed as mean ± S.D. (n = 3).

Superoxide radicals are generated in phenazine methosulfate-nicotinamide adenine dinucleotide (PMS/NADH) system by the reduction of nitroblue tetrazolium (NBT). For cold-water extracts, *G. frondosa* T1 and T2 showed scavenging abilities of 33.4% and 47.1% at 0.5 mg/mL, respectively. Scavenging abilities of *G. frondosa* T1 and T2 hot-water extracts were 56.9% and 62 at 0.5 mg/mL, respectively. In general, the scavenging abilities of superoxide radicals for both extracts were in the descending order of *G. frondosa* T2 hot-water > *G. frondosa* T1 hot-water > *G. frondosa* T2 cold-water > *G. frondosa* T1 cold-water extracts. However, the scavenging ability of L-ascorbic acid was up to 85% at 1 mg/mL, whereas the scavenging abilities of superoxide radicals of ethanolic extracts from both *G. frondosa* T1 and T2 were not detected (not shown in Figure 4). Song and Yen [19] demonstrated that the superoxide scavenging abilities were 40.2% and 30.0% for water and methanol extracts of mycelia from *Antrodia camphorata *at 200 µg/mL, respectively. Superoxide anions indirectly initiate lipid oxidation as superoxides and hydrogen peroxides serve as the precursors of singlet oxygen and hydroxyl radicals. As the result, both *G. frondosa* T1 and T2 water extracts may be the potential scavengers of superoxide anions to prevent lipid oxidation.

### 2.6. EC_50_ Values in Antioxidant Properties

Antioxidant properties investigated in this study are expressed as EC_50_ values for comparison (Table 1). Higher EC_50_ values indicate lower effectiveness in antioxidant properties. Effectiveness in reducing power was in a descending order of cold-water > ethanolic > hot-water extracts. As to the scavenging ability on DPPH radicals, various extracts were effective in the order of ethanolic > hot-water > cold-water extracts. The ethanolic extract with EC_50_ of 5.51 mg/mL was more effective than that of hot-water extract (17.36 mg/mL) and cold-water extract (19.42 mg/mL) from *G. frondosa* T1. Effectiveness in chelating ability on ferrous ions was in a descending order of cold-water > hot-water > ethanolic extracts. EC_50 _values in chelating effect on ferrous ions were 0.88, 2.33, and 4.82 mg/mL for cold-water extract, hot-water extract, and ethanolic extract from *G. frondosa* T1, respectively. The three extracts of *G. frondosa* T1 showed dominant chelating effects on ferrous ions, and the similar trend was found in three extracts of *G. frondosa* T2.

As to superoxide radical scavenging, the effectiveness was in a descending order of hot-water > cold-water extracts with *G. frondosa* T2 being more effective than T1. EC_50_ values in scavenging ability on superoxide radical were 0.95 and 0.62 mg/mL for cold-water extracts and 0.07 and 0.01 for hot-water extracts from *G. frondosa* T1 and T2, respectively. These results suggest that both extracts exhibited good scavenging superoxide radical activities as evidenced by their low EC_50_ values (*<*1 mg/mL).

In general, EC_50_ values of the three extracts of *G. frondosa* T1 and T2 were below 20 mg/mL for all antioxidant properties investigated in this study. Accordingly, the extracts of *G. frondosa* T1 and T2 exhibited effective antioxidant properties. These results imply that extracts of *G. frondosa* T1 and T2 may have potential benefit to protect human beings from oxidative damage.

### 2.7. Antioxidant Components

Several potential antioxidant components including total phenols, flavonoids, tocopherols, ascorbic acid and β-carotene were determined (Table 2). Ascorbic acid was mainly found in water extracts from *G. frondosa* T1 (2.90–5.45 mg/mL) and T2 (5.60–5.76 mg/mL). However, β-carotene was not detected in water extracts. The *α*-tocopherol was mainly found in ethanolic extracts, and the content of tocopherols in ethanolic extracts from *G. frondosa* T1 and T2, were 24.38 and 3.87 mg/g, respectively, which was considerably high in mushrooms reported previously.

**Table 1:** EC_50_ values of various extracts from *Grifola frondosa *T1 (A) and T2 (B) for antioxidant properties.

	EC_50_ values (mg/mL) *					
	(A)	T1		(B)	T2	
	Ethanolic	Cold water	Hot water	Ethanolic	Cold water	Hot water
Reducing power **	2.43 ± 0.01a	0.37 ± 0.04b	3.15 ± 0.01c	1.88 ± 0.00a	0.99 ± 0.01b	2.74 ± 0.01c
Scavenging effect on DPPH radicals **	3.51 ± 0.02a	19.42 ± 0.00b	17.36 ± 0.01c	5.07 ± 0.02a	10.25 ± 0.02b	9.27 ± 0.00c
Chelating effect on ferrous ions **	4.82 ± 0.02a	0.88 ± 0.08b	2.33 ± 0.05c	12.07 ± 0.09a	1.06 ± 0.03b	1.88 ± 0.01c
Scavenging ability on superoxide anion **	ND ***	0.95 ± 0.01a	0.07 ± 0.00b	ND	0.62 ± 0.02a	0.01 ± 0.00b

^*^ EC_50_ value represents the effective concentration at which the absorbance being 0.5 for reducing power, the DPPH scavenging effect being 50%, the superoxide anion scavenging ability being 50% or the ferrous ions chelating effect being 50%. EC_50_ value was obtained by interpolation from linear regression analysis. ^**^ Each value is expressed as mean ± SD (n = 3). Means with different letters of various extracts from the same strain of *Grifola frondosa* are significantly different (*P* < 0.05). ^*** ^Non-detectable.

**Table 2:** Antioxidant contents of extracts from *Grifola frondosa* T1 (A) and T2 (B).

	Contents (mg/g) *					
	(A)	T1		(B)	T2	
	Ethanolic	Cold water	Hot water	Ethanolic	Cold water	Hot water
Total phenols **	19.61 ± 1.69a	39.78 ± 1.86b	30.78 ± 0.42c	15.48 ± 1.78a	38.96 ± 1.14b	42.30 ± 1.64c
Flavonoid **	3.05 ± 0.10a	1.09 ± 0.06b	1.19 ± 0.05c	0.11 ± 0.03a	0.52 ± 0.04b	0.76 ± 0.04c
Ascorbic acid **	ND ***	5.45 ± 0.40a	2.90 ± 0.35b	ND	5.76 ± 0.43a	5.60 ± 0.25b
α-Tocopherol **	24.38 ± 1.98a	2.82±0.38b	ND	3.87 ± 0.21	ND	ND
β-Carotene **	ND	ND	ND	ND	ND	ND

^*^ Each value is expressed as mean ± standard deviation (n = 3). ^**^ Means with different letters Means with different letters of various extracts from the same strain of *Grifola frondosa* are significantly different (*P* < 0.05). ^*** ^Non-detectable.

Flavonoids act as antioxidant agents by direct free radical scavenging, transition metal chelation and maintenance of endogenous antioxidants, such as glutathione and superoxide dismutase systems. Total flavonoid content was 1.09–3.05 mg/g and 0.11–0.76 mg/g for *G. frondosa* T1 and T2, respectively.

Phenols are known to be the effective antioxidants in plants due to their hydroxyl groups. Typical phenols with antioxidant activity have been characterized as phenolic acids and flavonoids. Phenolic acids are repeatedly implicated as natural antioxidants in fruits, vegetables and other plants [16]. Total phenols were found in three extracts of *G. frondosa* T1 (19.61–39.78 mg/g) or T2 (15.48–42.30 mg/g). Water extracts contained more total phenols than ethanolic extracts did. The results from this study were similar to the results reported by Lee *et al*. [11]. In addition, there was a strong correlation between the contents of total phenols and EC_50_ values of chelating ferrous ions ability with the correlation coefficients (*R*^2^) being 0.992 and 0.968 for *G. frondosa* T1 and T2, respectively.

Polyphenols, such as BHT and gallate, are known to be the effective antioxidants. The high content of total polyphenolics in* G. frondosa* T1 and T2 may be the key components accounting for the high reducing power, metal chelating activity and superoxide anion scavenging ability. Numerous studies have conclusively shown that consumption of foods high in phenolic content reduce the risk of heart disease [16]. However, other specific active components that may also contribute in part to the antioxidant properties of these extracts from *G. frondosa* T1 and T2, and will require further isolation and identification.

### 2.8. β-Glucan Content

According to the published literature, the polysaccharide purified from maitake mushroom extracts had very strong anti-tumor capabilities [3]. Clinical studies had demonstrated that this glucan activates macrophages, subsequently increasing T-cell cascade to elevate the immune defense mechanism in the body [3]. However, β-(1→3)-D-glucan with β-(1→6) branched chain had been proved to have the greatest anti-tumor effect [3]. The β-glucan is used in enhancing defense mechanisms by activating macrophages, natural killer cells and T lymphocytes in immune system to promote the body resistance to the tumors. For *G. frondosa* T1, the ethanolic extract β-glucan content, 13.25 ± 1.38%, was slightly higher than that of the hot-water extract (10.69 ± 0.82% glucan) and the cold-water extract (10.04 ± 1.39% glucan). The results of the *G. frondosa* T2 extracts indicated that the ethanolic extract, containing 18.73 ± 1.68% β-glucan, was significantly higher (*P *< 0.05) than that of the cold-water extract (9.26 ± 0.30% glucan) and hot-water extract (10.26 ± 0.06% glucan). Polysaccharides extracted from several mushrooms had reported to contain antioxidant properties by their free radical scavenging ability [21]. *G. frondosa* T1 and T2 extracts contained polysaccharides that may contribute in part to the antioxidant properties.

## 3. Experimental

### 3.1. Mushroom Fruit Bodies

The ground powders of dried mushroom fruit bodies of *G. frondosa* T1 and T2 strains were obtained from the Agricultural Research Institute, Taichung, Taiwan. For each of ethanolic, cold and hot water extractions from *G. frondosa* T1 and T2, three dried samples were randomly selected and prepared for analyses. For ethanolic extraction, a subsample (10 g) was extracted by stirring with 95% ethanol (100 mL) at RT for 24 h, then filtering through Whatman Number 1 filter paper. The residue was further extracted with two additional volumes of 100 mL ethanol as described above. The combined extracts were rotary evaporated at 40 °C to dryness and the resulting filtrate was freeze-dried. For cold-water extraction, a subsample (10 g) was extracted by stirring with cold water (100 mL) at 4 °C for 24 h and filtering through Whatman Number 1 filter paper. The residue was further extracted with two additional volumes of 100 mL cold water as described above. The combined cold-water extracts were rotary evaporated at 40 °C to dryness and the resulting filtrate was freeze-dried. For hot-water extraction, a subsample (10 g) was heated with deionized water (100 mL) at reflux for 3 h and filtered through Whatman Number 1 filter paper. The residue was further extracted with two additional volumes of boiling water (100 mL) as described above. The combined hot-water extracts were rotary evaporated at 40 °C to dryness and the resulting filtrate was freeze-dried. The extraction yield is calculated according to the following formula: extraction yield = (extract weight/fruiting body dry weight) × 100%.

### 3.2. Reducing Power

The reducing power was determined following the method described by Oyaizu [22]. Each extract (1–20 mg/mL) in deionized water (2.5 mL) was mixed with 0.2 M sodium phosphate buffer (2.5 mL, pH 6.6, Riedel-de-Haën and J.T. Baker) and 1% potassium ferricyanide (2.5 mL, Riedel-de-Haën), and the mixture was incubated at 50 °C for 20 min. After adding 10% trichloroacetic acid (2.5 mL, w/v, Riedel-de-Haën), the mixture was centrifuged at 200 × g for 10 min. The upper layer (5 mL) was mixed with deionized water (5 mL) and 0.1% ferric chloride (1 mL, Riedel-de-Haën), and the absorbance was measured at 700 nm. The higher absorbance indicates higher reducing power. Ascorbic acid and butylated hydroxytoluene (BHT) were used as controls. The EC_50_ value (mg extract/mL), the effective concentration at which the absorbance being 0.5 for reducing power, was obtained by interpolation from linear regression analysis. 

### 3.3. Scavenging Ability on 1,1-Diphenyl-2-Picrylhydrazyl (DPPH) Radicals

Each extract (1–20 mg/mL) in deionized water (4 mL) was mixed with methanolic solution (1 mL) containing DPPH (Sigma), resulting in a final concentration of 0.2 mM DPPH. The mixture was shaken vigorously and left to stand for 30 min in the dark. DPPH radical reduction was determined by measuring the absorbance at 517 nm [23]. Scavenging ability (%) = [(Δ*A*_517_ of control − Δ*A*_517_ of sample)/Δ*A*_517_ of control] × 100%. Ascorbic acid and BHT were used as controls. The EC_50_ value(mg extract/mL), the effective concentration at which the DPPH scavenging effect being 50%, was obtained by interpolation from linear regression analysis.

### 3.4. Chelating Abilities on Ferrous Ions

Chelating ability was determined according to the method of Dinis *et al*. [24]. Each extract (0.1–20 mg/mL) in water (1 mL) was mixed with methanol (3.7 mL) and 2 mM ferrous chloride (0.1 mL, Fluka). The reaction was initiated by addition of 5 mM ferrozine (0.2 mL, Sigma). After 10 min at RT, the absorbance of the mixture was determined at 562 nm. Chelating ability (%) = [(Δ*A*_562_ of control − Δ*A*_562_ of sample)/Δ*A*_562_ of control] × 100%. Ethylenediaminetetraacetic acid (EDTA, Sigma) was used as controls. The EC_50_ value (mg extract/mL), the effective concentration at which the ferrous ions chelating effect being 50%, was obtained by interpolation from linear regression analysis. 

### 3.5. Scavenging Ability on Superoxide Anion

Scavenging ability was determined according to the method of Robak and Gryglewski [25]. Each extract (0.01–1 mg/mL) in water (1 mL) was mixed with 80 μM phenazine methosulfate (PMS, 1 mL, Sigma), dihydronicotinamide adenine dinucleotide (NADH, 624 μM) and nitro blue tetrazolium (NBT, 200 μM, Sigma) in 0.1 M sodium phosphate buffer (pH 7.4), and then incubated at RT for 5 min. The absorbance was measured at 560 nm and ascorbic acid was used for comparison. Scavenging capability to superoxide radicals = [(*A*_560_ of control − *A*_560_ of sample)/*A*_560_ of control] × 100%. Ascorbic acid and BHT were used as controls. The EC_50_ value (mg extract/mL), the effective concentration at which the scavenging superoxide anion activity being 50%, was obtained by interpolation from linear regression analysis.

### 3.6. Determination of Antioxidant Components

Total phenols were determined according to the method of Taga *et al*. [26]. The total phenol content was calculated with the calibration curve of gallic acid (Sigma). Total flavonoid content was determined by the colorimetric method of Bao *et al*. [27] with minor modification. A total of 0.5 mL of appropriately diluted extracts or standard solutions were pipetted into 15 mL polypropylene conical tubes containing double distilled H_2_O (2 mL) and mixed with 5% NaNO_2_ (0.15 mL, Merck). After 5 min, 10% AlCl_3_ 6H_2_O solution (0.15 mL, Merck) was added and the mixture was allowed to stand for additional 5 min, and then 1 M NaOH (1 mL) was added. The reaction solution was mixed well and kept for 15 min. Absorbance of the mixture was measured at 415 nm. Total flavonoid content was calculated using the standard quercetin (Sigma) curve, and expressed as milligram quercetin equivalent per gram of dry weight.

Ascorbic acid was determined according to the method of Klein and Perry [28]. The content of ascorbic acid was estimated by the calibration curve of authentic L-ascobic acid.

β-Carotene was extracted and analyzed as described by Rundhang *et al*. [29]. Dried extract (100 mg) was further extracted with ethanol (1 mL), *n*-hexane (2 mL) containing butylated hydroxyanisole (BHA, 25 μg/mL) and deionized water (1 mL), mixed for 1 min at RT, and then centrifuged at 400 × g for 10 min. After the removal of the *n*-hexane layer with N_2_, the volume was adjusted to 1 mL using *n*-hexane and then filtered through a 0.45-μm cellulose acetate (CA) filter before being injected into a high-performance liquid chromatograph (HPLC). The HPLC system consisted of a Jasco 2086 plus pump, a Jasco 2075 plus UV-Vis detector, a Jasco AS-2055 autosampler, a Jasco MX-2080-31 solvent mixing module, a ERC-3215α degaser and a Waters Sunfire 100 RP-18 (4.6 × 250 mm, 5 μm) column. The mobile phase was methanol/toluene (3:1, v/v) at a flow rate of 0.4 mL/min and UV detection was at 450 nm. Content of β-carotene was estimated by the calibration curve of authentic β-carotene.

The α-tocopherols were extracted and analyzed according to the method of Carpenter [30]. The organic layer was washed with deionized water to neutral, dried over anhydrous sodium sulfate and rotary evaporated to dry completely. The residue was redissolved in *n*-hexane (5 mL) and filtered prior to HPLC injection. The HPLC system used for α-tocopherols assay was the same as that for β-carotene assay. The mobile phase was 85 mL acetonitrile/15 mL methanol at a flow rate of 0.6 mL/min and UV detection was at 295 nm. Content of α-tocopherol was estimated by the calibration curve of α-tocopherol (Sigma). The β-glucan content was analyzed using a β-glucan assay kit (Megazyme, Ireland).

### 3.7. Statistical Analysis

The mean values and the standard deviation were from the data of triplicate trials. Mean values were compared by analysis of variance (ANOVA) with Duncan’s multiple range method for comparing groups [31]. A significance level of 5% was adopted for all comparisons.

## 4. Conclusions

The results from this study conclusively demonstrate the antioxidant activities of three extracts from *G. frondosa* T1 and T2. Various extracts were examined for radical-scavenging activity by DPPH, scavenging ability on superoxide anion, chelating ability on ferrous ions and reducing power. Based on the results obtained in this study, the three extracts of *G. frondosa* T1 and T2 mushroom species have significant *in vitro* antioxidant activity against various oxidant systems. The total phenols are associated with the EC_50_ value for chelating ferrous ions. Therefore, the extracts from *G. frondosa* T1 and T2 may be the good sources for antioxidative-related functional foods and the pharmaceutical industry. Furthermore, *G. frondosa* can be used as an easily accessible source of natural antioxidants and as a possible food supplement. The various antioxidant mechanisms of *G. frondosa *extracts may be attributed in part by strong hydrogen-donating ability, metal-chelating ability and the effectiveness as good scavengers of superoxide and free radicals. For application in the food industry, the fractionation of three extracts and further identification of active components merit further investigation.
